# Dabigatran etexilate tetra­hydrate

**DOI:** 10.1107/S1600536812046673

**Published:** 2012-11-17

**Authors:** Hong-Qiang Liu, Wei-Guang Zhang, Zhi-Qiang Cai, Wei-Ren Xu, Xiu-Ping Shen

**Affiliations:** a State Key Laboratory of Drug Delivery Technology and Pharmacokinetics, Tianjin Centre for New Drug Safety Assessment and Research, Tianjin 300193, People’s Republic of China; b Graduate School, Tianjin University of Traditional Chinese Medicine, Tianjin 300193, People’s Republic of China

## Abstract

In the title compound, C_34_H_41_N_7_O_5_·4H_2_O (systematic name: ethyl 3-{[2-({4-[(*Z*)-amino­(hexyl­oxycarbonyl­imino)­meth­yl]anilino}meth­yl)-1-meth­yl­benzimidazole-5-carbon­yl]pyridin-2-yl­amino}­propano­ate tetra­hydrate), the benzene and pyridine rings form dihedral angles of 5.4 (1) and 43.8 (1)°, respectively, with the benzimidazole mean plane. The terminal butyl group is disordered over two conformations in a 0.756 (10):0.244 (10) ratio. There is an intramolecular N—H⋯O hydrogen bond present. In the crystal, the water mol­ecules are involved in the formation of O—H⋯O, O—H⋯N and N—H⋯O hydrogen bonds, which link the components into layers parallel to the *ab* plane.

## Related literature

For background to the anti­coagulant drug dabigatran etexil­ate, see: Nagarakanti & Ellis (2012 ▶); Van Ryn *et al.* (2010 ▶).
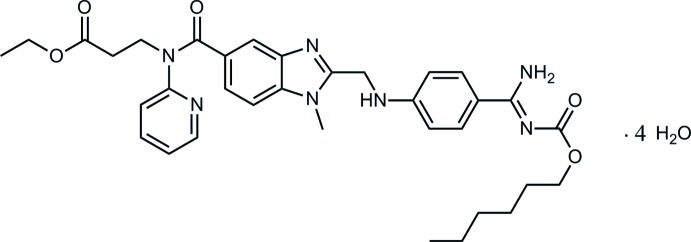


## Experimental

### 

#### Crystal data

C_34_H_41_N_7_O_5_·4H_2_O
*M* *_r_* = 699.80
Triclinic, 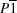

*a* = 9.1140 (13) Å
*b* = 10.9700 (14) Å
*c* = 18.3830 (17) Å
α = 88.51 (1)°
β = 85.455 (9)°
γ = 83.034 (12)°
*V* = 1818.4 (4) Å^3^
*Z* = 2
Mo *K*α radiation
μ = 0.09 mm^−1^
*T* = 113 K
0.22 × 0.20 × 0.18 mm

#### Data collection

Rigaku Saturn 724 CCD area-detector diffractometer
Absorption correction: multi-scan (*CrystalClear*; Rigaku/MSC, 2009 ▶) *T*_min_ = 0.980, *T*_max_ = 0.983
23569 measured reflections
8627 independent reflections
5187 reflections with *I* > 2σ(*I*)
*R*_int_ = 0.036

#### Refinement

*R*[*F* ^2^ > 2σ(*F* ^2^)] = 0.039
*wR*(*F* ^2^) = 0.103
*S* = 0.96
8627 reflections
513 parameters
40 restraints
H atoms treated by a mixture of independent and constrained refinement
Δρ_max_ = 0.42 e Å^−3^
Δρ_min_ = −0.37 e Å^−3^

### 

Data collection: *CrystalClear* (Rigaku/MSC, 2009 ▶); cell refinement: *CrystalClear*; data reduction: *CrystalClear*; program(s) used to solve structure: *SHELXS97* (Sheldrick, 2008 ▶); program(s) used to refine structure: *SHELXL97* (Sheldrick, 2008 ▶); molecular graphics: *SHELXTL* (Sheldrick, 2008 ▶); software used to prepare material for publication: *SHELXTL*.

## Supplementary Material

Crystal structure: contains datablock(s) I, global. DOI: 10.1107/S1600536812046673/cv5357sup1.cif

Structure factors: contains datablock(s) I. DOI: 10.1107/S1600536812046673/cv5357Isup2.hkl

Supplementary material file. DOI: 10.1107/S1600536812046673/cv5357Isup3.cml

Additional supplementary materials:  crystallographic information; 3D view; checkCIF report

## Figures and Tables

**Table 1:** Hydrogen-bond geometry (Å, °)

*D*—H⋯*A*	*D*—H	H⋯*A*	*D*⋯*A*	*D*—H⋯*A*
N1—H1⋯O7^i^	0.896 (17)	1.980 (18)	2.8468 (16)	162.5 (15)
N1—H2⋯O1	0.902 (15)	1.931 (16)	2.6281 (16)	132.7 (13)
O6—H8⋯N5	0.912 (19)	1.88 (2)	2.7938 (16)	176.3 (16)
O6—H16⋯O3^ii^	0.84 (2)	2.00 (2)	2.8373 (15)	172.1 (18)
O7—H17⋯O8	0.861 (19)	1.954 (19)	2.8061 (16)	170.2 (18)
O7—H18⋯O9	0.881 (18)	1.871 (19)	2.7513 (15)	176.5 (17)
O9—H20⋯N2	0.93 (2)	2.00 (2)	2.9226 (16)	168.8 (19)
O9—H20⋯O2	0.93 (2)	2.43 (2)	3.1126 (14)	129.6 (16)
O9—H24⋯N7^iii^	0.844 (19)	2.050 (19)	2.8918 (16)	175.5 (18)
O8—H25⋯O6	0.84 (2)	2.04 (2)	2.8689 (17)	171 (2)
O8—H32⋯O6^iii^	0.94 (2)	1.89 (2)	2.8291 (17)	175.3 (17)

Symmetry codes: (i) 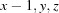; (ii) 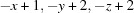; (iii) 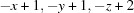.

**Fig. 1.**
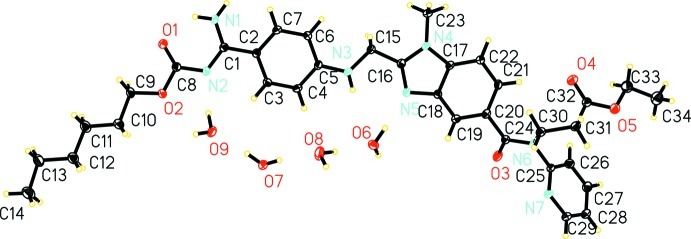
View of the title compound showing the atomic numbering and 40% probability displacement ellipsoids. For the disordered atoms, only major parts are shown.

## supplementary crystallographic information

### Comment

Dabigatran etexilate is an oral anticoagulant drug (Nagarakanti & Ellis 2012; Van Ryn*et al.*, 2010). During our study on this drug, we obtained the single crystals of its tetrahydrate, which provides a valuable insight into the crystal structure of this drug.

In the title compound (Fig. 1), the benzene and pyridine rings form dihedral angles of 5.4 (1) and 43.8 (1)°, respectively, with the benzimidazole mean plane. The terminal butyl group is disordered over two conformations in a ratio 0.756 (1):0.244 (1). The crystalline water molecules are involved in formation of O—H···O, O—H···N and N—H···O hydrogen bonds (Table 1), which link all moieties into layers parallel to the *ab* plane.

### Experimental

5.00 g (10 mmol) of ethyl 3-(2-((4-carbamimidoylphenylamino)methyl)-1-methyl-N-(pyridin-2-yl)- 1*H*-benzo[*d*]imidazole-5-carboxamido)propanoate was dissolved in 40 ml of THF and 10 ml of water, and 8.29 (60 mmol) of potassium carbonate was added. The mixture was stirred at room temperature for 20 min, followed by dropwise addition of 3.29 g (20 mmol) of n-hexyl chloroformate. The reaction mixture was stirred at room temperature until the reaction completed (typical 4–6 h). The reaction mixture was diluted with 100 ml of dichloromethane, washed with saturated brine, dried over sodium sulfate and evaporated on a rotary evaporator to afford the crude product as a white solid. The crude product was triturated with ethyl acetate/petroleum ether (1/2) to yield the pure product as a white solid. Single crystals suitable for X-ray diffraction were obtained through slow evaporation of a solution of the pure title compound in ethyl acetate/petroleum ether (1/2 by volume).

### Refinement

N- and O-bound H atoms were located on a difference map and isotropically refined. C-bound H atoms were geometrically positioned (C–H = 0.95–0.99 Å), and included in the final cycles of refinement using a riding model, with *U*_iso_(H) = 1.2–1.5*U*_eq_(C).

### Crystal data

**Table:**

C_34_H_41_N_7_O_5_·4H_2_O	*Z* = 2
*M**_r_* = 699.80	*F*(000) = 748
Triclinic, *P*1	*D*_x_ = 1.278 Mg m^−^^3^
Hall symbol: -P 1	Mo *K*α radiation, λ = 0.71073 Å
*a* = 9.1140 (13) Å	Cell parameters from 6648 reflections
*b* = 10.9700 (14) Å	θ = 1.9–27.9°
*c* = 18.3830 (17) Å	µ = 0.09 mm^−^^1^
α = 88.51 (1)°	*T* = 113 K
β = 85.455 (9)°	Prism, colourless
γ = 83.034 (12)°	0.22 × 0.20 × 0.18 mm
*V* = 1818.4 (4) Å^3^	

### Data collection

**Table:**

Rigaku Saturn 724 CCD area-detector diffractometer	8627 independent reflections
Radiation source: rotating anode	5187 reflections with *I* > 2σ(*I*)
Multilayer monochromator	*R*_int_ = 0.036
Detector resolution: 14.222 pixels mm^-1^	θ_max_ = 27.9°, θ_min_ = 1.9°
ω and φ scans	*h* = −11→11
Absorption correction: multi-scan (*CrystalClear*; Rigaku/MSC, 2009)	*k* = −14→14
*T*_min_ = 0.980, *T*_max_ = 0.983	*l* = −24→24
23569 measured reflections	

### Refinement

**Table:**

Refinement on *F*^2^	Secondary atom site location: difference Fourier map
Least-squares matrix: full	Hydrogen site location: inferred from neighbouring sites
*R*[*F*^2^ > 2σ(*F*^2^)] = 0.039	H atoms treated by a mixture of independent and constrained refinement
*wR*(*F*^2^) = 0.103	*w* = 1/[σ^2^(*F*_o_^2^) + (0.0522*P*)^2^] where *P* = (*F*_o_^2^ + 2*F*_c_^2^)/3
*S* = 0.96	(Δ/σ)_max_ = 0.003
8627 reflections	Δρ_max_ = 0.42 e Å^−^^3^
513 parameters	Δρ_min_ = −0.37 e Å^−^^3^
40 restraints	Extinction correction: *SHELXL97* (Sheldrick, 2008), Fc^*^=kFc[1+0.001xFc^2^λ^3^/sin(2θ)]^-1/4^
Primary atom site location: structure-invariant direct methods	Extinction coefficient: 0.0080 (11)

### Special details

**Table:**

Geometry. All e.s.d.'s (except the e.s.d. in the dihedral angle between two l.s. planes) are estimated using the full covariance matrix. The cell e.s.d.'s are taken into account individually in the estimation of e.s.d.'s in distances, angles and torsion angles; correlations between e.s.d.'s in cell parameters are only used when they are defined by crystal symmetry. An approximate (isotropic) treatment of cell e.s.d.'s is used for estimating e.s.d.'s involving l.s. planes.
Refinement. Refinement of *F*^2^ against ALL reflections. The weighted *R*-factor *wR* and goodness of fit *S* are based on *F*^2^, conventional *R*-factors *R* are based on *F*, with *F* set to zero for negative *F*^2^. The threshold expression of *F*^2^ > σ(*F*^2^) is used only for calculating *R*-factors(gt) *etc*. and is not relevant to the choice of reflections for refinement. *R*-factors based on *F*^2^ are statistically about twice as large as those based on *F*, and *R*- factors based on ALL data will be even larger.

### Fractional atomic coordinates and isotropic or equivalent isotropic displacement parameters (Å^2^)

**Table:**

	*x*	*y*	*z*	*U*_iso_*/*U*_eq_	Occ. (<1)
O1	−0.07658 (10)	0.08373 (9)	0.62025 (5)	0.0267 (2)	
O2	0.17115 (10)	0.05322 (9)	0.62048 (5)	0.0270 (2)	
O3	0.42751 (11)	1.14550 (9)	1.09974 (5)	0.0287 (2)	
O4	0.04154 (11)	1.24401 (10)	1.31026 (6)	0.0381 (3)	
O5	0.17441 (10)	1.34627 (9)	1.37972 (5)	0.0279 (2)	
O6	0.39167 (12)	0.67342 (10)	0.95328 (6)	0.0275 (2)	
O7	0.50826 (11)	0.30578 (10)	0.79165 (6)	0.0295 (3)	
O8	0.44104 (13)	0.41395 (12)	0.92879 (7)	0.0384 (3)	
O9	0.35290 (12)	0.12591 (10)	0.74559 (6)	0.0312 (3)	
N1	−0.20452 (13)	0.22911 (11)	0.72416 (7)	0.0235 (3)	
N2	0.05361 (12)	0.18903 (10)	0.69881 (6)	0.0203 (3)	
N3	0.03535 (13)	0.61137 (11)	0.93156 (7)	0.0240 (3)	
N4	−0.10668 (11)	0.85922 (10)	1.05945 (6)	0.0183 (2)	
N5	0.12518 (11)	0.79155 (10)	1.01673 (6)	0.0192 (2)	
N6	0.35894 (11)	1.13593 (10)	1.22064 (6)	0.0189 (2)	
N7	0.60387 (12)	1.13966 (10)	1.25116 (6)	0.0224 (3)	
C1	−0.06690 (14)	0.24665 (12)	0.73487 (7)	0.0181 (3)	
C2	−0.04330 (14)	0.33887 (11)	0.78872 (7)	0.0176 (3)	
C3	0.09907 (14)	0.37209 (12)	0.79453 (7)	0.0203 (3)	
H3	0.1804	0.3318	0.7653	0.024*	
C4	0.12344 (14)	0.46122 (12)	0.84135 (7)	0.0212 (3)	
H4	0.2212	0.4814	0.8443	0.025*	
C5	0.00592 (14)	0.52322 (12)	0.88503 (7)	0.0187 (3)	
C6	−0.13680 (14)	0.49039 (12)	0.88028 (7)	0.0215 (3)	
H6	−0.2180	0.5307	0.9095	0.026*	
C7	−0.15985 (14)	0.39941 (12)	0.83304 (7)	0.0213 (3)	
H7	−0.2571	0.3777	0.8307	0.026*	
C8	0.03660 (15)	0.10830 (12)	0.64545 (7)	0.0206 (3)	
C9	0.17173 (15)	−0.03820 (13)	0.56542 (8)	0.0270 (3)	
H9A	0.1270	−0.0005	0.5216	0.032*	
H9B	0.1133	−0.1043	0.5842	0.032*	
C10	0.33034 (16)	−0.09023 (14)	0.54624 (8)	0.0337 (4)	
H10A	0.3766	−0.1134	0.5905	0.040*	0.756 (10)
H10B	0.3820	−0.0275	0.5222	0.040*	0.756 (10)
H10C	0.3709	−0.1281	0.5891	0.040*	0.244 (10)
H10D	0.3863	−0.0240	0.5312	0.040*	0.244 (10)
C11	0.3481 (5)	−0.1909 (4)	0.4920 (3)	0.0363 (10)	0.756 (10)
H11A	0.3006	−0.1595	0.4475	0.044*	0.756 (10)
H11B	0.2939	−0.2585	0.5125	0.044*	0.756 (10)
C12	0.5069 (4)	−0.2433 (3)	0.4700 (2)	0.0317 (8)	0.756 (10)
H12A	0.5580	−0.2657	0.5148	0.038*	0.756 (10)
H12B	0.5578	−0.1782	0.4440	0.038*	0.756 (10)
C13	0.5237 (4)	−0.3553 (4)	0.4216 (2)	0.0413 (9)	0.756 (10)
H13A	0.4765	−0.4224	0.4476	0.050*	0.756 (10)
H13B	0.4724	−0.3345	0.3766	0.050*	0.756 (10)
C14	0.6862 (7)	−0.3987 (8)	0.4014 (5)	0.0448 (5)	0.756 (10)
H14A	0.7345	−0.3306	0.3788	0.067*	0.756 (10)
H14B	0.6941	−0.4663	0.3669	0.067*	0.756 (10)
H14C	0.7347	−0.4273	0.4454	0.067*	0.756 (10)
C11'	0.3396 (14)	−0.2118 (12)	0.5051 (9)	0.0363 (10)	0.244 (10)
H11C	0.2894	−0.2720	0.5360	0.044*	0.244 (10)
H11D	0.2871	−0.1973	0.4600	0.044*	0.244 (10)
C12'	0.4992 (14)	−0.2644 (12)	0.4854 (7)	0.0317 (8)	0.244 (10)
H12C	0.5631	−0.1990	0.4909	0.038*	0.244 (10)
H12D	0.5270	−0.3304	0.5213	0.038*	0.244 (10)
C13'	0.5337 (14)	−0.3157 (13)	0.4106 (6)	0.0413 (9)	0.244 (10)
H13C	0.5348	−0.2470	0.3746	0.050*	0.244 (10)
H13D	0.4542	−0.3646	0.3993	0.050*	0.244 (10)
C14'	0.683 (2)	−0.397 (2)	0.4025 (14)	0.0448 (5)	0.244 (10)
H14D	0.7121	−0.4104	0.3506	0.067*	0.244 (10)
H14E	0.6737	−0.4758	0.4274	0.067*	0.244 (10)
H14F	0.7582	−0.3559	0.4243	0.067*	0.244 (10)
C15	−0.08019 (14)	0.68470 (12)	0.97473 (7)	0.0200 (3)	
H15A	−0.1329	0.6308	1.0090	0.024*	
H15B	−0.1528	0.7268	0.9423	0.024*	
C16	−0.01657 (14)	0.77783 (12)	1.01663 (7)	0.0183 (3)	
C17	−0.01616 (14)	0.93177 (12)	1.09055 (7)	0.0179 (3)	
C18	0.12911 (14)	0.88895 (11)	1.06367 (7)	0.0174 (3)	
C19	0.24838 (14)	0.94473 (12)	1.08399 (7)	0.0194 (3)	
H19	0.3469	0.9177	1.0655	0.023*	
C20	0.21816 (14)	1.04163 (12)	1.13237 (7)	0.0194 (3)	
C21	0.07151 (15)	1.08313 (12)	1.15878 (7)	0.0210 (3)	
H21	0.0545	1.1496	1.1917	0.025*	
C22	−0.04793 (15)	1.02964 (12)	1.13805 (7)	0.0209 (3)	
H22	−0.1468	1.0583	1.1554	0.025*	
C23	−0.26885 (14)	0.86874 (13)	1.07118 (8)	0.0249 (3)	
H23A	−0.3103	0.8480	1.0261	0.030*	
H23B	−0.3081	0.9529	1.0848	0.030*	
H23C	−0.2964	0.8117	1.1104	0.030*	
C24	0.34361 (14)	1.10995 (12)	1.14951 (7)	0.0201 (3)	
C25	0.47803 (14)	1.20529 (12)	1.23399 (7)	0.0189 (3)	
C26	0.45451 (15)	1.33195 (12)	1.23265 (8)	0.0236 (3)	
H26	0.3627	1.3745	1.2200	0.028*	
C27	0.56949 (15)	1.39514 (13)	1.25036 (8)	0.0267 (3)	
H27	0.5577	1.4824	1.2504	0.032*	
C28	0.70211 (15)	1.32896 (13)	1.26806 (8)	0.0259 (3)	
H28	0.7828	1.3700	1.2800	0.031*	
C29	0.71403 (15)	1.20250 (13)	1.26791 (7)	0.0244 (3)	
H29	0.8047	1.1575	1.2803	0.029*	
C30	0.30221 (15)	1.06478 (12)	1.28412 (7)	0.0219 (3)	
H30A	0.3766	0.9940	1.2941	0.026*	
H30B	0.2105	1.0322	1.2722	0.026*	
C31	0.26932 (15)	1.14206 (13)	1.35196 (7)	0.0244 (3)	
H31A	0.2390	1.0891	1.3935	0.029*	
H31B	0.3611	1.1749	1.3636	0.029*	
C32	0.14955 (15)	1.24727 (14)	1.34383 (7)	0.0250 (3)	
C33	0.06161 (17)	1.45193 (14)	1.37596 (9)	0.0335 (4)	
H33A	−0.0344	1.4315	1.3990	0.040*	
H33B	0.0483	1.4764	1.3245	0.040*	
C34	0.11330 (19)	1.55442 (14)	1.41568 (9)	0.0388 (4)	
H34A	0.1276	1.5286	1.4663	0.047*	
H34B	0.0388	1.6267	1.4150	0.047*	
H34C	0.2073	1.5749	1.3917	0.047*	
H1	−0.2851 (19)	0.2647 (16)	0.7500 (10)	0.050 (5)*	
H2	−0.2158 (17)	0.1769 (14)	0.6885 (9)	0.033 (4)*	
H5	0.1212 (17)	0.6292 (14)	0.9290 (8)	0.034 (5)*	
H8	0.307 (2)	0.7148 (16)	0.9744 (10)	0.055 (6)*	
H16	0.442 (2)	0.7259 (17)	0.9336 (10)	0.057 (6)*	
H17	0.482 (2)	0.3315 (17)	0.8352 (11)	0.059 (6)*	
H18	0.455 (2)	0.2495 (17)	0.7780 (10)	0.053 (6)*	
H20	0.263 (3)	0.145 (2)	0.7243 (12)	0.085 (7)*	
H24	0.367 (2)	0.0486 (17)	0.7441 (10)	0.051 (6)*	
H25	0.431 (2)	0.4908 (19)	0.9311 (11)	0.068 (7)*	
H32	0.502 (2)	0.3853 (17)	0.9665 (11)	0.061 (6)*	

### Atomic displacement parameters (Å^2^)

**Table:**

	*U*^11^	*U*^22^	*U*^33^	*U*^12^	*U*^13^	*U*^23^
O1	0.0211 (5)	0.0330 (6)	0.0271 (5)	−0.0033 (4)	−0.0044 (4)	−0.0115 (4)
O2	0.0199 (5)	0.0321 (6)	0.0289 (5)	−0.0010 (4)	−0.0004 (4)	−0.0144 (5)
O3	0.0310 (6)	0.0362 (6)	0.0221 (5)	−0.0181 (5)	0.0004 (4)	−0.0038 (4)
O4	0.0240 (6)	0.0475 (7)	0.0435 (7)	0.0025 (5)	−0.0132 (5)	−0.0163 (6)
O5	0.0264 (5)	0.0280 (6)	0.0297 (6)	−0.0008 (4)	−0.0064 (4)	−0.0042 (5)
O6	0.0232 (6)	0.0238 (6)	0.0345 (6)	−0.0029 (5)	0.0040 (5)	−0.0018 (5)
O7	0.0228 (5)	0.0303 (6)	0.0367 (7)	−0.0089 (5)	0.0025 (5)	−0.0116 (5)
O8	0.0460 (7)	0.0293 (7)	0.0413 (7)	0.0022 (6)	−0.0193 (6)	−0.0113 (6)
O9	0.0274 (6)	0.0215 (6)	0.0468 (7)	−0.0018 (5)	−0.0143 (5)	−0.0088 (5)
N1	0.0188 (6)	0.0264 (7)	0.0262 (7)	−0.0043 (5)	−0.0017 (5)	−0.0116 (6)
N2	0.0185 (6)	0.0215 (6)	0.0212 (6)	−0.0016 (5)	−0.0024 (5)	−0.0059 (5)
N3	0.0166 (6)	0.0257 (7)	0.0309 (7)	−0.0055 (5)	−0.0013 (5)	−0.0107 (5)
N4	0.0158 (5)	0.0206 (6)	0.0196 (6)	−0.0059 (5)	−0.0013 (4)	−0.0028 (5)
N5	0.0189 (6)	0.0190 (6)	0.0205 (6)	−0.0036 (5)	−0.0030 (5)	−0.0022 (5)
N6	0.0169 (6)	0.0213 (6)	0.0200 (6)	−0.0065 (5)	−0.0029 (4)	−0.0028 (5)
N7	0.0189 (6)	0.0220 (6)	0.0268 (6)	−0.0024 (5)	−0.0039 (5)	−0.0039 (5)
C1	0.0186 (7)	0.0169 (7)	0.0190 (7)	−0.0029 (5)	−0.0023 (5)	0.0010 (5)
C2	0.0191 (7)	0.0160 (6)	0.0180 (6)	−0.0027 (5)	−0.0033 (5)	0.0003 (5)
C3	0.0168 (6)	0.0220 (7)	0.0213 (7)	0.0005 (6)	−0.0012 (5)	−0.0027 (6)
C4	0.0151 (6)	0.0236 (7)	0.0258 (7)	−0.0052 (6)	−0.0032 (5)	−0.0016 (6)
C5	0.0206 (7)	0.0167 (7)	0.0195 (7)	−0.0034 (5)	−0.0042 (5)	−0.0020 (5)
C6	0.0179 (7)	0.0214 (7)	0.0248 (7)	−0.0014 (6)	0.0004 (6)	−0.0069 (6)
C7	0.0170 (7)	0.0218 (7)	0.0261 (7)	−0.0054 (6)	−0.0026 (6)	−0.0028 (6)
C8	0.0204 (7)	0.0207 (7)	0.0207 (7)	−0.0032 (6)	0.0005 (6)	−0.0012 (6)
C9	0.0268 (8)	0.0300 (8)	0.0240 (7)	0.0000 (6)	−0.0027 (6)	−0.0123 (6)
C10	0.0250 (8)	0.0403 (9)	0.0347 (9)	0.0004 (7)	0.0018 (7)	−0.0148 (7)
C11	0.0340 (10)	0.0424 (17)	0.031 (2)	0.0088 (11)	−0.0096 (11)	−0.0134 (16)
C12	0.0310 (10)	0.0351 (15)	0.0277 (18)	0.0004 (11)	−0.0002 (11)	−0.0059 (12)
C13	0.0365 (11)	0.039 (2)	0.0472 (16)	0.0086 (16)	−0.0096 (10)	−0.0161 (16)
C14	0.0372 (10)	0.0537 (13)	0.0410 (11)	0.0068 (8)	−0.0023 (8)	−0.0127 (9)
C11'	0.0340 (10)	0.0424 (17)	0.031 (2)	0.0088 (11)	−0.0096 (11)	−0.0134 (16)
C12'	0.0310 (10)	0.0351 (15)	0.0277 (18)	0.0004 (11)	−0.0002 (11)	−0.0059 (12)
C13'	0.0365 (11)	0.039 (2)	0.0472 (16)	0.0086 (16)	−0.0096 (10)	−0.0161 (16)
C14'	0.0372 (10)	0.0537 (13)	0.0410 (11)	0.0068 (8)	−0.0023 (8)	−0.0127 (9)
C15	0.0202 (7)	0.0187 (7)	0.0221 (7)	−0.0051 (6)	−0.0025 (5)	−0.0027 (6)
C16	0.0203 (7)	0.0172 (7)	0.0179 (6)	−0.0036 (6)	−0.0024 (5)	0.0007 (5)
C17	0.0186 (7)	0.0179 (7)	0.0182 (6)	−0.0059 (5)	−0.0031 (5)	0.0006 (5)
C18	0.0186 (6)	0.0159 (6)	0.0179 (6)	−0.0024 (5)	−0.0020 (5)	−0.0006 (5)
C19	0.0165 (6)	0.0208 (7)	0.0213 (7)	−0.0031 (5)	−0.0029 (5)	0.0002 (6)
C20	0.0215 (7)	0.0190 (7)	0.0189 (7)	−0.0063 (6)	−0.0043 (5)	0.0012 (5)
C21	0.0240 (7)	0.0194 (7)	0.0204 (7)	−0.0041 (6)	−0.0022 (6)	−0.0044 (6)
C22	0.0174 (7)	0.0237 (7)	0.0218 (7)	−0.0030 (6)	−0.0010 (5)	−0.0042 (6)
C23	0.0173 (7)	0.0318 (8)	0.0269 (8)	−0.0078 (6)	0.0000 (6)	−0.0059 (6)
C24	0.0184 (7)	0.0197 (7)	0.0225 (7)	−0.0027 (6)	−0.0020 (5)	−0.0031 (6)
C25	0.0163 (6)	0.0223 (7)	0.0187 (7)	−0.0040 (6)	−0.0019 (5)	−0.0041 (6)
C26	0.0209 (7)	0.0221 (7)	0.0277 (8)	−0.0009 (6)	−0.0041 (6)	−0.0007 (6)
C27	0.0283 (8)	0.0196 (7)	0.0332 (8)	−0.0063 (6)	−0.0031 (6)	−0.0028 (6)
C28	0.0221 (7)	0.0271 (8)	0.0303 (8)	−0.0080 (6)	−0.0044 (6)	−0.0057 (6)
C29	0.0176 (7)	0.0284 (8)	0.0281 (8)	−0.0037 (6)	−0.0051 (6)	−0.0040 (6)
C30	0.0203 (7)	0.0235 (7)	0.0229 (7)	−0.0060 (6)	−0.0028 (6)	−0.0001 (6)
C31	0.0211 (7)	0.0318 (8)	0.0213 (7)	−0.0061 (6)	−0.0042 (6)	−0.0006 (6)
C32	0.0203 (7)	0.0345 (8)	0.0209 (7)	−0.0067 (6)	−0.0001 (6)	−0.0049 (6)
C33	0.0331 (9)	0.0330 (9)	0.0335 (9)	0.0036 (7)	−0.0078 (7)	−0.0015 (7)
C34	0.0444 (10)	0.0295 (9)	0.0431 (10)	−0.0031 (8)	−0.0084 (8)	−0.0015 (8)

### Geometric parameters (Å, º)

**Table:**

O1—C8	1.2251 (15)	C12—H12A	0.9900
O2—C8	1.3510 (15)	C12—H12B	0.9900
O2—C9	1.4425 (15)	C13—C14	1.520 (5)
O3—C24	1.2316 (16)	C13—H13A	0.9900
O4—C32	1.2071 (16)	C13—H13B	0.9900
O5—C32	1.3367 (16)	C14—H14A	0.9800
O5—C33	1.4571 (17)	C14—H14B	0.9800
O6—H8	0.912 (19)	C14—H14C	0.9800
O6—H16	0.84 (2)	C11'—C12'	1.519 (9)
O7—H17	0.861 (19)	C11'—H11C	0.9900
O7—H18	0.881 (18)	C11'—H11D	0.9900
O8—H25	0.84 (2)	C12'—C13'	1.495 (9)
O8—H32	0.94 (2)	C12'—H12C	0.9900
O9—H20	0.93 (2)	C12'—H12D	0.9900
O9—H24	0.844 (19)	C13'—C14'	1.530 (9)
N1—C1	1.3223 (16)	C13'—H13C	0.9900
N1—H1	0.896 (17)	C13'—H13D	0.9900
N1—H2	0.902 (15)	C14'—H14D	0.9800
N2—C1	1.3354 (16)	C14'—H14E	0.9800
N2—C8	1.3688 (16)	C14'—H14F	0.9800
N3—C5	1.3713 (16)	C15—C16	1.4928 (17)
N3—C15	1.4426 (17)	C15—H15A	0.9900
N3—H5	0.826 (15)	C15—H15B	0.9900
N4—C16	1.3593 (16)	C17—C22	1.3901 (17)
N4—C17	1.3780 (15)	C17—C18	1.4054 (17)
N4—C23	1.4680 (16)	C18—C19	1.3904 (17)
N5—C16	1.3188 (16)	C19—C20	1.3914 (18)
N5—C18	1.3963 (15)	C19—H19	0.9500
N6—C24	1.3669 (16)	C20—C21	1.4109 (18)
N6—C25	1.4386 (16)	C20—C24	1.4985 (17)
N6—C30	1.4810 (17)	C21—C22	1.3809 (17)
N7—C25	1.3337 (17)	C21—H21	0.9500
N7—C29	1.3438 (16)	C22—H22	0.9500
C1—C2	1.4794 (17)	C23—H23A	0.9800
C2—C7	1.3975 (18)	C23—H23B	0.9800
C2—C3	1.4023 (17)	C23—H23C	0.9800
C3—C4	1.3685 (17)	C25—C26	1.3798 (18)
C3—H3	0.9500	C26—C27	1.3881 (18)
C4—C5	1.4036 (18)	C26—H26	0.9500
C4—H4	0.9500	C27—C28	1.390 (2)
C5—C6	1.4010 (18)	C27—H27	0.9500
C6—C7	1.3854 (17)	C28—C29	1.3784 (19)
C6—H6	0.9500	C28—H28	0.9500
C7—H7	0.9500	C29—H29	0.9500
C9—C10	1.5064 (19)	C30—C31	1.5159 (18)
C9—H9A	0.9900	C30—H30A	0.9900
C9—H9B	0.9900	C30—H30B	0.9900
C10—C11	1.494 (3)	C31—C32	1.502 (2)
C10—C11'	1.540 (9)	C31—H31A	0.9900
C10—H10A	0.9601	C31—H31B	0.9900
C10—H10B	0.9600	C33—C34	1.498 (2)
C10—H10C	0.9601	C33—H33A	0.9900
C10—H10D	0.9601	C33—H33B	0.9900
C11—C12	1.519 (3)	C34—H34A	0.9800
C11—H11A	0.9900	C34—H34B	0.9800
C11—H11B	0.9900	C34—H34C	0.9800
C12—C13	1.522 (4)		
			
C8—O2—C9	116.08 (10)	C13'—C12'—C11'	116.6 (9)
C32—O5—C33	115.53 (11)	C13'—C12'—H12C	108.1
H8—O6—H16	107.3 (16)	C11'—C12'—H12C	108.1
H17—O7—H18	113.2 (17)	C13'—C12'—H12D	108.1
H25—O8—H32	105.7 (17)	C11'—C12'—H12D	108.1
H20—O9—H24	103.2 (16)	H12C—C12'—H12D	107.3
C1—N1—H1	124.5 (10)	C12'—C13'—C14'	112.8 (9)
C1—N1—H2	116.5 (10)	C12'—C13'—H13C	109.0
H1—N1—H2	119.1 (14)	C14'—C13'—H13C	109.0
C1—N2—C8	119.06 (11)	C12'—C13'—H13D	109.0
C5—N3—C15	122.41 (11)	C14'—C13'—H13D	109.0
C5—N3—H5	116.9 (10)	H13C—C13'—H13D	107.8
C15—N3—H5	120.0 (10)	C13'—C14'—H14D	109.5
C16—N4—C17	106.66 (10)	C13'—C14'—H14E	109.5
C16—N4—C23	127.23 (11)	H14D—C14'—H14E	109.5
C17—N4—C23	126.11 (11)	C13'—C14'—H14F	109.5
C16—N5—C18	104.75 (10)	H14D—C14'—H14F	109.5
C24—N6—C25	116.67 (10)	H14E—C14'—H14F	109.5
C24—N6—C30	124.33 (10)	N3—C15—C16	110.61 (11)
C25—N6—C30	115.31 (10)	N3—C15—H15A	109.5
C25—N7—C29	117.00 (12)	C16—C15—H15A	109.5
N1—C1—N2	124.48 (12)	N3—C15—H15B	109.5
N1—C1—C2	118.39 (12)	C16—C15—H15B	109.5
N2—C1—C2	117.10 (11)	H15A—C15—H15B	108.1
C7—C2—C3	117.59 (11)	N5—C16—N4	113.56 (11)
C7—C2—C1	122.34 (11)	N5—C16—C15	125.99 (12)
C3—C2—C1	120.02 (11)	N4—C16—C15	120.45 (11)
C4—C3—C2	121.45 (12)	N4—C17—C22	131.62 (12)
C4—C3—H3	119.3	N4—C17—C18	105.80 (10)
C2—C3—H3	119.3	C22—C17—C18	122.57 (11)
C3—C4—C5	120.90 (12)	C19—C18—N5	130.40 (12)
C3—C4—H4	119.6	C19—C18—C17	120.36 (11)
C5—C4—H4	119.6	N5—C18—C17	109.23 (10)
N3—C5—C6	122.64 (12)	C18—C19—C20	117.59 (12)
N3—C5—C4	119.02 (12)	C18—C19—H19	121.2
C6—C5—C4	118.33 (11)	C20—C19—H19	121.2
C7—C6—C5	120.21 (12)	C19—C20—C21	121.15 (11)
C7—C6—H6	119.9	C19—C20—C24	118.17 (12)
C5—C6—H6	119.9	C21—C20—C24	120.35 (11)
C6—C7—C2	121.51 (12)	C22—C21—C20	121.72 (12)
C6—C7—H7	119.2	C22—C21—H21	119.1
C2—C7—H7	119.2	C20—C21—H21	119.1
O1—C8—O2	120.94 (11)	C21—C22—C17	116.59 (12)
O1—C8—N2	129.76 (12)	C21—C22—H22	121.7
O2—C8—N2	109.30 (11)	C17—C22—H22	121.7
O2—C9—C10	107.93 (11)	N4—C23—H23A	109.5
O2—C9—H9A	110.1	N4—C23—H23B	109.5
C10—C9—H9A	110.1	H23A—C23—H23B	109.5
O2—C9—H9B	110.1	N4—C23—H23C	109.5
C10—C9—H9B	110.1	H23A—C23—H23C	109.5
H9A—C9—H9B	108.4	H23B—C23—H23C	109.5
C11—C10—C9	114.1 (2)	O3—C24—N6	121.11 (11)
C11—C10—C11'	12.7 (8)	O3—C24—C20	120.02 (12)
C9—C10—C11'	111.3 (5)	N6—C24—C20	118.82 (11)
C11—C10—H10A	113.1	N7—C25—C26	124.33 (12)
C9—C10—H10A	108.8	N7—C25—N6	115.85 (11)
C11'—C10—H10A	103.9	C26—C25—N6	119.71 (12)
C11—C10—H10B	103.7	C25—C26—C27	117.78 (13)
C9—C10—H10B	108.9	C25—C26—H26	121.1
C11'—C10—H10B	115.7	C27—C26—H26	121.1
H10A—C10—H10B	107.9	C26—C27—C28	119.05 (13)
C11—C10—H10C	104.4	C26—C27—H27	120.5
C9—C10—H10C	108.9	C28—C27—H27	120.5
C11'—C10—H10C	94.3	C29—C28—C27	118.55 (13)
H10A—C10—H10C	10.7	C29—C28—H28	120.7
H10B—C10—H10C	117.0	C27—C28—H28	120.7
C11—C10—H10D	112.7	N7—C29—C28	123.29 (13)
C9—C10—H10D	108.7	N7—C29—H29	118.4
C11'—C10—H10D	124.1	C28—C29—H29	118.4
H10A—C10—H10D	98.3	N6—C30—C31	112.12 (11)
H10B—C10—H10D	10.7	N6—C30—H30A	109.2
H10C—C10—H10D	107.9	C31—C30—H30A	109.2
C10—C11—C12	115.4 (3)	N6—C30—H30B	109.2
C10—C11—H11A	108.4	C31—C30—H30B	109.2
C12—C11—H11A	108.4	H30A—C30—H30B	107.9
C10—C11—H11B	108.4	C32—C31—C30	113.14 (11)
C12—C11—H11B	108.4	C32—C31—H31A	109.0
H11A—C11—H11B	107.5	C30—C31—H31A	109.0
C11—C12—C13	115.1 (3)	C32—C31—H31B	109.0
C11—C12—H12A	108.5	C30—C31—H31B	109.0
C13—C12—H12A	108.5	H31A—C31—H31B	107.8
C11—C12—H12B	108.5	O4—C32—O5	123.06 (14)
C13—C12—H12B	108.5	O4—C32—C31	124.96 (13)
H12A—C12—H12B	107.5	O5—C32—C31	111.97 (11)
C14—C13—C12	110.9 (4)	O5—C33—C34	107.38 (12)
C14—C13—H13A	109.5	O5—C33—H33A	110.2
C12—C13—H13A	109.5	C34—C33—H33A	110.2
C14—C13—H13B	109.5	O5—C33—H33B	110.2
C12—C13—H13B	109.5	C34—C33—H33B	110.2
H13A—C13—H13B	108.0	H33A—C33—H33B	108.5
C12'—C11'—C10	111.6 (9)	C33—C34—H34A	109.5
C12'—C11'—H11C	109.3	C33—C34—H34B	109.5
C10—C11'—H11C	109.3	H34A—C34—H34B	109.5
C12'—C11'—H11D	109.3	C33—C34—H34C	109.5
C10—C11'—H11D	109.3	H34A—C34—H34C	109.5
H11C—C11'—H11D	108.0	H34B—C34—H34C	109.5
			
C8—N2—C1—N1	1.9 (2)	C23—N4—C17—C18	179.88 (12)
C8—N2—C1—C2	−176.19 (12)	C16—N5—C18—C19	−179.11 (14)
N1—C1—C2—C7	6.0 (2)	C16—N5—C18—C17	−0.32 (14)
N2—C1—C2—C7	−175.86 (12)	N4—C17—C18—C19	179.02 (12)
N1—C1—C2—C3	−171.36 (12)	C22—C17—C18—C19	−0.2 (2)
N2—C1—C2—C3	6.82 (19)	N4—C17—C18—N5	0.08 (14)
C7—C2—C3—C4	−0.6 (2)	C22—C17—C18—N5	−179.18 (12)
C1—C2—C3—C4	176.89 (13)	N5—C18—C19—C20	179.84 (13)
C2—C3—C4—C5	−0.4 (2)	C17—C18—C19—C20	1.16 (19)
C15—N3—C5—C6	−5.5 (2)	C18—C19—C20—C21	−1.1 (2)
C15—N3—C5—C4	175.89 (12)	C18—C19—C20—C24	−174.58 (12)
C3—C4—C5—N3	179.58 (13)	C19—C20—C21—C22	0.1 (2)
C3—C4—C5—C6	0.9 (2)	C24—C20—C21—C22	173.45 (13)
N3—C5—C6—C7	−179.07 (13)	C20—C21—C22—C17	0.8 (2)
C4—C5—C6—C7	−0.4 (2)	N4—C17—C22—C21	−179.80 (13)
C5—C6—C7—C2	−0.5 (2)	C18—C17—C22—C21	−0.7 (2)
C3—C2—C7—C6	1.0 (2)	C25—N6—C24—O3	−0.57 (19)
C1—C2—C7—C6	−176.37 (13)	C30—N6—C24—O3	−157.83 (13)
C9—O2—C8—O1	−2.42 (19)	C25—N6—C24—C20	−177.95 (11)
C9—O2—C8—N2	177.92 (11)	C30—N6—C24—C20	24.79 (19)
C1—N2—C8—O1	5.4 (2)	C19—C20—C24—O3	48.24 (19)
C1—N2—C8—O2	−174.95 (11)	C21—C20—C24—O3	−125.29 (15)
C8—O2—C9—C10	−178.74 (12)	C19—C20—C24—N6	−134.35 (13)
O2—C9—C10—C11	177.3 (3)	C21—C20—C24—N6	52.12 (18)
O2—C9—C10—C11'	163.8 (8)	C29—N7—C25—C26	−0.2 (2)
C9—C10—C11—C12	178.5 (2)	C29—N7—C25—N6	−176.35 (11)
C11'—C10—C11—C12	−101 (3)	C24—N6—C25—N7	−95.43 (15)
C10—C11—C12—C13	173.3 (5)	C30—N6—C25—N7	63.90 (15)
C11—C12—C13—C14	178.7 (5)	C24—N6—C25—C26	88.23 (15)
C11—C10—C11'—C12'	75 (3)	C30—N6—C25—C26	−112.44 (14)
C9—C10—C11'—C12'	179.8 (9)	N7—C25—C26—C27	0.0 (2)
C10—C11'—C12'—C13'	−137.5 (15)	N6—C25—C26—C27	176.00 (12)
C11'—C12'—C13'—C14'	−164.4 (16)	C25—C26—C27—C28	0.4 (2)
C5—N3—C15—C16	−176.57 (12)	C26—C27—C28—C29	−0.5 (2)
C18—N5—C16—N4	0.46 (15)	C25—N7—C29—C28	0.0 (2)
C18—N5—C16—C15	−179.85 (13)	C27—C28—C29—N7	0.3 (2)
C17—N4—C16—N5	−0.41 (15)	C24—N6—C30—C31	−153.22 (12)
C23—N4—C16—N5	179.89 (12)	C25—N6—C30—C31	49.24 (15)
C17—N4—C16—C15	179.87 (12)	N6—C30—C31—C32	62.82 (15)
C23—N4—C16—C15	0.2 (2)	C33—O5—C32—O4	0.0 (2)
N3—C15—C16—N5	−1.54 (19)	C33—O5—C32—C31	−178.75 (12)
N3—C15—C16—N4	178.13 (12)	C30—C31—C32—O4	36.1 (2)
C16—N4—C17—C22	179.36 (14)	C30—C31—C32—O5	−145.15 (12)
C23—N4—C17—C22	−0.9 (2)	C32—O5—C33—C34	−176.98 (12)
C16—N4—C17—C18	0.18 (14)		

### Hydrogen-bond geometry (Å, º)

**Table:**

*D*—H···*A*	*D*—H	H···*A*	*D*···*A*	*D*—H···*A*
N1—H1···O7^i^	0.896 (17)	1.980 (18)	2.8468 (16)	162.5 (15)
N1—H2···O1	0.902 (15)	1.931 (16)	2.6281 (16)	132.7 (13)
O6—H8···N5	0.912 (19)	1.88 (2)	2.7938 (16)	176.3 (16)
O6—H16···O3^ii^	0.84 (2)	2.00 (2)	2.8373 (15)	172.1 (18)
O7—H17···O8	0.861 (19)	1.954 (19)	2.8061 (16)	170.2 (18)
O7—H18···O9	0.881 (18)	1.871 (19)	2.7513 (15)	176.5 (17)
O9—H20···N2	0.93 (2)	2.00 (2)	2.9226 (16)	168.8 (19)
O9—H20···O2	0.93 (2)	2.43 (2)	3.1126 (14)	129.6 (16)
O9—H24···N7^iii^	0.844 (19)	2.050 (19)	2.8918 (16)	175.5 (18)
O8—H25···O6	0.84 (2)	2.04 (2)	2.8689 (17)	171 (2)
O8—H32···O6^iii^	0.94 (2)	1.89 (2)	2.8291 (17)	175.3 (17)

Symmetry codes: (i) *x*−1, *y*, *z*; (ii) −*x*+1, −*y*+2, −*z*+2; (iii) −*x*+1, −*y*+1, −*z*+2.
